# Integrating empathic and mentalizing abilities with interpersonal sensitivity in people with eating disorders: A network analysis approach

**DOI:** 10.1192/j.eurpsy.2021.950

**Published:** 2021-08-13

**Authors:** G. Patriciello, L. Marone, A. Vece, E. Barone, A.M. Monteleone

**Affiliations:** Psychiatry, University of Campania “Luigi Vanvitelli”, napoli, Italy

**Keywords:** eating disorders, interpersonal sensitivity, Network analysis, social cognition

## Abstract

**Introduction:**

Literature highlights that interpersonal sensitivity represents an important development and maintaining factor for Eating Disorder (ED). Mentalizing and empathy are two psychological constructs that play a crucial role in social functioning. However, the role of mentalizing and empathy in the socio-emotional processing deficits of ED patients has been under investigated.

**Objectives:**

We aimed to assess the complex interactions between the sub-components of mentalizing and empathy and ED symptoms through a network analysis approach.

**Methods:**

Seventy-seven women with EDs were included in our study. Eating disorder and affective symptomatology were investigated with self-report questionnaires. All patients underwent two computerized tasks: Movie for the Assessment of Social Cognition (MASC), assessing emotional and non-emotional mental state inferences; Empathic Accuracy Task-Revised (EAT-R), measuring accuracy in identifying and sharing others’ emotions. A partial correlation network and bridge function analyses were computed.

**Results:**

In the partial correlation network inference of cognitive mental states and shape concern were the nodes with the highest strength centrality. Inference of emotional mental states was the node with the highest bridge strength in the cluster of social cognition functions. Empathic and mentalizing abilities were directly connected with each other and with ED symptoms.

**Conclusions:**

This is the first network analysis study which integrates self-reported symptoms and objective socio-cognitive performance in people with Eds. Our results provide evidence of the complex interactions between mentalizing, empathy and psychopathological symptoms in people with EDs. Therefore, confirm that the ability to infer others’ mental state may represent a useful target for clinical intervention in EDs.

